# Immediate and late effects of long-term testicular heat stress on the number of seminiferous tubules and cellular content in Santa Inês rams

**DOI:** 10.1590/1984-3143-AR2024-0134

**Published:** 2025-10-20

**Authors:** Luana Vanessa Ribeiro, Bárbara Rost Dalchiavon, Mayra Elena Ortiz D´Ávila Assumpção, Thais Rose dos Santos Hamilton

**Affiliations:** 1 Departamento de Morfologia e Fisiologia Animal, Faculdade de Ciências Agrárias e Veterinárias, Universidade Estadual Paulista “Júlio de Mesquita Filho” – UNESP, Jaboticabal, SP, Brasil; 2 Departamento de Reprodução Animal, Faculdade de Medicina Veterinária e Zootecnia, Universidade de São Paulo – USP, São Paulo, SP, Brasil

**Keywords:** spermatogenesis, testicular insulation, spermatogonia, spermatocyte, orchiectomy

## Abstract

Efficient spermatogenesis in mammals occurs when testicular temperature is approximately 2 to 8 °C below body temperature. Elevated testicular temperature can trigger oxidative stress and compromise sperm integrity during spermatogenesis, potentially resulting in damaged spermatozoa and male infertility. This study aimed to evaluate how heat stress affects the quantity of seminiferous tubules, and the abundance of germ cells within the seminiferous tubules. To this end, six Santa Inês rams were subjected to testicular insulation for 12 consecutive days, followed by two hemi-orchiectomies, the first 24 hours after insulation period to evaluate the immediate effect, and the second 30 days after the first hemi-orchiectomy to evaluate the late effect. Six Santa Inês rams composed the control group. Histological analyses were conducted to quantify the number of seminiferous tubules and the types of cells within them (spermatogonia, spermatocytes, and spermatids) in testicular fragments. Despite an increase in testicular temperature, no significant differences were observed in the number of seminiferous tubules. These findings probably reflect the resistance of Santa Ines rams to high environment temperatures. Regarding the abundance of cells, a decrease in spermatogonia (0.27% ± 0.06; 0.05% ± 0.03*, p* = 0.005) and an increase in spermatocytes (35.90% ± 1.58; 46.77% ± 4.33*, p* = 0.028) were observed immediately after the insulation period compared to 30 days after, the late effect. This result suggests an effect of the first hemi-orchiectomy on the remaining testicle, probably an attempt to maintain sperm production.

## Introduction

Sperm are produced within seminiferous tubules through a cyclic, synchronized, and continuous process called spermatogenesis. These small, coiled tubules containing germinative epithelium, surrounded by loose connective tissue with high vascularization, nerves, and interstitial cells (Valencise et al., 2021) that produce testosterone.

The final number of seminiferous tubules in testicle is reached during the early embryo development in mammals, when sexual differentiation determines the individual characteristics of the gonads based on sexual chromosome. The Y chromosome triggers the testicle differentiation through the SRY gene (sex-determining region Y) by action of the proteins and transcription factors modulate by Testicular Differentiation Factor (Reviello, 2024). Transcription factors such as SF-1 and WT-1 play critical roles in Sertoli and Leydig cells to produce anti-Müllerian hormone (AMH), which induces the regression of paramesonephric ducts (Moore et al., 2013). Another key transcription factor is SOX9, essential throughout male development, that in its absence, determine development of the female characteristics, involving multiple ovarian-determining genes and inhibitors of testicular formation (Gonen et al., 2017). Additionally, SOX9 works with SF-1 to regulate AMH production (Ming et al., 2022). Collectively, these molecular mechanisms underpin the formation and structural development of the testes, including the definitive number of seminiferous tubules established during the embryogenesis (Reviello, 2024).

Following testicular development, the sperm production called spermatogenesis, that occurs inside the seminiferous tubules, remains dormant until the onset of puberty. At this stage, the spermatogonia, the undifferentiated germ cell, resume proliferation increasing in number and size through mitotic divisions, undergoing morphological modifications to produce the primary spermatocytes. Primary spermatocytes undergo reductive division (first meiosis) to produce secondary spermatocytes that complete the second meiotic division to produce haploids spermatids. The final phase, spermiogenesis, involves the morphological transformation of spermatids into mature spermatozoa, characterized by nuclear condensation, cytoplasmic remodeling and elimination, acrosome and flagellum development. Mature spermatozoa are then released into the lumen of the seminiferous tubule and transported to the epididymis for maturation and storage (Moore et al., 2013).

The efficiency of spermatogenesis in mammals is highly dependent of the maintenance of testicular temperature below body temperature. Several anatomical and physiological adaptations contribute to this thermoregulation. The mechanisms involving the scrotum, testicular vascular cone (including the pampiniform plexus), smooth muscle in the scrotum (tunica dartos), and the cremaster muscle, that acts to suspend the testicle, regulate testicular temperature to maintain the scrotal temperature between 2 and 8^o^ C below body temperature (Senger, 2005). Heat dissipation occurs through the arrangement of testicular arteries and veins in the pampiniform plexus, where a blood countercurrent mechanism balances blood flow temperature within the testis (Almeida et al., 2008).

Exposure to elevated testicular temperatures disrupts this balance and cause heat stress by the increase of the testicular metabolism while maintain constant blood supply (Hamilton et al., 2016; Paul et al., 2009). This mechanism results in reduced oxygen flow to tissues, ischemia, and subsequent hypoxia (Zhang et al., 2024). However, there are recent evidence that changes in testicular metabolism are due to hyperthermia *per se* (Rizzoto et al., 2018), and even under acute hypoxic conditions, the testicles are capable of maintain oxygen delivery and uptake by increasing blood flow and oxygen extraction, with no evidence of hypoxia.

Nevertheless, increased blood flow potentially induced by hypoxia would increase testicular temperature and metabolism, which would lead to oxidative stress. Oxidative stress is defined as an imbalance between the production of reactive oxygen species (ROS) and the capacity of the antioxidant molecules to neutralize the reactive molecules (Walczak-Jedrzejowska et al., 2013). When the antioxidant capacity of the testicular becomes overwhelmed, cellular components including lipids, proteins and nucleic acids, may suffer severe damage (Saleh and Agarwal, 2002).

While physiological levels of ROS are crucial for sperm viability, maturation and capacitation (Dutta et al., 2021); excessive ROS production under heat stress induces detrimental effects. Testicular heat stress induced sperm chromatin and DNA damage (Hamilton et al., 2016; Hamilton et al., 2018; Hamilton and Assumpção, 2020). Moreover, oxidative stress impairs sperm morphology and functionality due to subcellular damage, potentially resulting in male infertility (Asadi et al., 2017). Hence, oxidative stress has been identified as the main mechanism underlying testicular damage during heat stress (Fleming et al., 2004; Nichi et al., 2006; Paul et al., 2009). Despite severe sperm damage by oxidative stress, the sperm capacity to fertilize oocytes may retain but embryo development could be compromised (Tsakmakidis, 2010).

A comprehensive review by Robinson et al. (2023) highlights the detrimental impacts of testicular heat stress on spermatogenesis and the importance of thermal regulation in male fertility.

Based on this evidence, the present study hypothesized that the long-term testicular heat stress compromises the number of seminiferous tubules and decrease the number of germinative cells within them. To test this hypothesis, twelve 8-month-old Santa Inês rams were randomly allocated into two groups: six subjected to 12 consecutive days (288 hours) of testicular heat stress (treated group) and six maintained under standard conditions (control group). Histological testicle evaluations were performed immediately and late (30 days) after testicular insulation period to assess the immediate and long-term effects of heat stress on the seminiferous tubules and the germinative cells within them.

## Methods

Twelve mature (8 months old) Santa Inês rams were used in a randomized experimental design with repeated measures over time. The animals were submitted to uniform nutritional conditions and kept in individual stalls. The experiment was approved by the Bioethics committee of the School of Veterinary Medicine and Animal Sciences, University of Sao Paulo (n^o^ 2445-2011). Before the experiment began, all animals underwent clinical and reproductive evaluations, confirming that none exhibited any pathology. Reproductive assessments, including motility, sperm concentration, mass motility, sperm morphology, and sperm membrane integrity, showed no significant differences (p > 0.10) among them. The animals were divided into two groups: six subjected to 12 consecutive days (288 hours) of testicular heat stress (treated group) and six controls (control group). In the treated group, rams underwent testicular heat stress using an insulating bag on the scrotum, designed to fully enclose the testicles, maintaining the temperature in scrotal region and ensuring uniform heat retention. A custom-designed prototype was developed to integrate thermal insulation principles with the specific conditions of individual stalls. The bag consisted of three distinct layers: an outer layer made of waterproof material; a middle layer composed of thermal insulating material (acrylic blanket) to minimize heat dissipation from the testicles to the environment; and an inner layer made of soft and non-abrasive material (felt) to prevent tissue irritation. Velcro strap was placed at the top and side to allow proper adjustment during application. Testicular temperatures were measured daily inserting a digital thermometer inside the insulating bag. The temperature in the testicles of the rams subjected to testicular heat stress was 33.29°C ± 0.34 and 28.05°C ± 1.30 in control group.

The prolonged testicular insulation period was established because the experimental phase was conducted during the Brazilian winter (July - August) and the environmental temperature was 17.01 ± 0.52°C, with a relative humidity of 78.08 ± 0.73%.

After the insulation period, to evaluate the immediate effect of the testicular heat stress was performed a hemi-orchiectomy 24 hours after the removal of insulation bag, with the removal of the left testis. The second hemi-orchiectomy was performed 30 days after the first orchiectomy with the removal the right testis to evaluate the late effect of the testicular heat stress. The experimental design considered the ram spermatic cycle (stages I and VIII) to ensure spermatids and spermatozoa in our histology samples.

The orchiectomies were carried out in accordance with the recommendations of the Brazilian Federal Council of Veterinary Medicine. All the twelve mature Santa Inês rams were sedated with Xylazine (0.02 mg/kg), and a local anesthetic (2% Lidocaine with epinephrine; 10 ml per testicle) were performed. An antibiotic (Ceftiofur 2.2 mg/kg, single dose) and a non-steroidal anti-inflammatory (Ketoprofen 3 mg/kg IV) were administered before and after the surgery. After scrotal antisepsis, hemi orchiectomy was performed using a scalpel, followed by clamping and twisting of the spermatic cord, with subsequent testicle removal.

**Figure 1:**
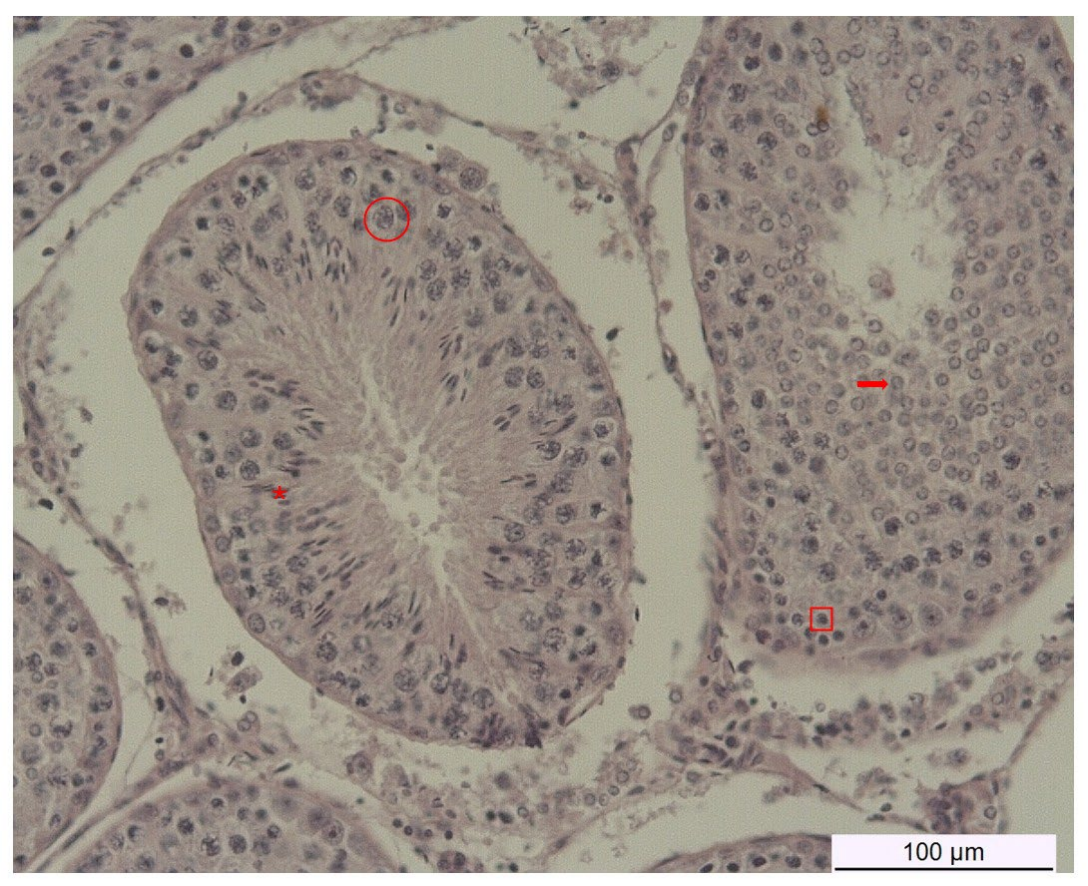
Histological image of the mature ram testicle indicating the different germ cells within the seminiferous tubules according to McMillan and Harris (2018). Legend - red square: spermatogonia; red circle: spermatocyte; red arrow: round spermatid; red asterisks: elongated spermatid.

Testicular fragments of approximately 30 mg were fixed in Metacarn solution [60% (v/v) methanol, 30% (v/v) chloroform, and 10% (v/v) glacial acetic acid]. After 24 hours, fragments were immersed in 95% ethanol (v/v) until processing and paraffin embedding. Paraffin-embedded testicular fragments were sectioned, mounted on slides, and subjected to deparaffinization and hydration. Slides were stained with Harris hematoxylin and eosin (HE). Histological sections were analyzed under a Axioplan 2 microscope with an Axiocam HRc camera (Zeiss Oberkochen, German). The total of seminiferous tubules was evaluated by the observation of ten random fields at 20x magnification for each experimental unit, group, and recovery time. The percentage of spermatogonia, spermatocytes, round and elongated spermatids within them were counted in ten seminiferous tubules at 50x magnification for each experimental unit, group, and recovery time. The identification of the different cell types was performed as described by McMillan and Harris (2018). Only seminiferous tubules with intact lumens, seminiferous epithelium, and interstitial spaces were considered eligible for evaluation (Figure 1). The histological assessment was performed in a blinded manner with respect to the experimental group and recovery time. The percentage of each cell type within individual seminiferous tubules was calculated relative to the total cell count.

JASP 0.19 software was used for data analysis. The ANOVA procedure was used to evaluate the model factorial 2x2 between experimental group (treated and control) and recovery time (immediate and late).

## Results

No statistical difference was observed in the number of seminiferous tubules for interaction effect between experimental groups X recovery time (p = 0.23; Table 1). No differences were observed in the number of seminiferous tubules for experimental group effect (p = 0.23; Table 2) and for recovery time effect (p = 0.38; Table 2), independently.

**Table 1:** Number of seminiferous tubules and the percentage of spermatogonia, spermatocyte, round and elongated spermatid in the ram seminiferous tubules for interaction effect between experimental groups (control and treated) and recovery time (immediate, 24 hours after the insulation period; and late, 30 days after the immediate time).

**Experimental Groups**	**Control**		**Treated**		***p***
**Recovery Time**	**Immediate**	**Late**	**Immediate**	**Late**	
Number of seminiferous tubules	271.50±37.84	278.33±27.41	351.50±37.97	282.75±33.56	0.28
Spermatogonia (%)	0.26±0.13	0.004±0.004	0.27±0.03	0.09±0.05	0.58
Spermatocyte (%)	37.34±2.92	46.96±8.71	34.45±1.27	46.59±2.60	0.79
Round Spermatid (%)	31.41±5.22	33.93±3.77	33.06±5.07	27.35±3.61	0.37
Elongated Spermatid (%)	31.49±5.10	28.93±2.44	32.20±4.39	25.95±3.15	0.64

Statistical significance (*p*). Values are expressed as mean ± standard error of the mean (SEM).

**Table 2:** Number of seminiferous tubules and the percentage of spermatogonia, spermatocyte, round and elongated spermatid inside of the ram seminiferous tubules. Columns 2, 3 and 4: in experimental groups (control x treated) independent of the recovery time, probability values (*p*). Columns 5, 6 and 7: in recovery time (immediate, 24 hours after the insulation period; and late, 30 days after the immediate time) independent of the experimental groups.

	**Experimental Groups**			**Recovery Time**		
	**Control**	**Treated**	***p***	**Immediate**	**Late**	***p***
**Seminiferous tubules**	274.91±22.30	317.12±26.29	**0.23**	311.50±28.26	280.54±20.66	**0.38**
**Spermatogonia (%)**	0.135±0.07	0.185±0.042	**0.56**	0.27±0.06^a^	0.05±0.03^b^	**0.005**
**Spermatocyte (%)**	42.15±4.61	40.52±2.29	**0.75**	35.90±1.58^a^	46.77±4.33^b^	**0.028**
**Round Spermatid (%)**	32.67±3.096	30.21±3.091	**0.58**	32.23±3.47	30.64±2.68	**0.72**
**Elongated Spermatid (%)**	30.21±2.72	29.08±2.74	**0.77**	31.85±3.21	0.05±0.03	**0.25**

Statistical significance (*p*). Values are expressed as mean ± standard error of the mean (SEM).

**Figure 2:**
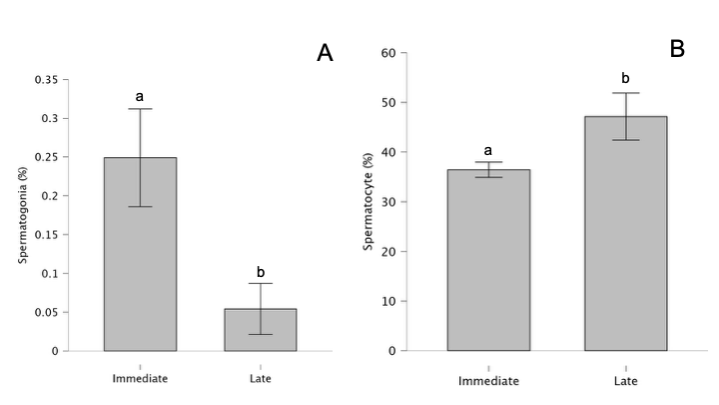
A: Spermatogonia per seminiferous tubule (%) and B: Spermatocyte per seminiferous tubule (%) in the recovery time (immediate: 24 hours after the insulation period; late – 30 days after the immediate time). Different letters indicate statistical difference.

Regarding the percentage of spermatogonia, spermatocytes, round spermatids, and elongated spermatids within the seminiferous tubules, no statistical difference was observed for interaction effect (Table 1) and for experimental group effect (treated group vs. control group) independent of the recovery time (Table 2). However, considering the recovery time effect independent of the experimental group, a decrease in the percentage of spermatogonia (Figure 2A) and an increase in the percentage of spermatocytes (Figure 2B) were observed at late compared to immediate effect (Table 2).

## Discussion

Despite the 6°C increase in testicular environmental temperature in treated compared to control group, the heat stress was not sufficient to alter the number of testicular seminiferous tubules or the abundance of spermatogonia, spermatocytes, round and elongated spermatids within them. The alteration in cellular abundance within seminiferous tubules submitted to heat stress was described by Hamilton et al. (2018) but the number of tubules appears to remain unaffected.

The final number of seminiferous tubules in the testicle is determined during early embryonic development in mammals, when sexual differentiation is driven by the Y chromosome (Reviello, 2024). Thus, even with the increase of the temperature caused by the heat stress performed in this study the number of seminiferous tubules remained unchanged.

Hamilton et al. (2018) observed in the rams submitted to heat stress histological features of initial testicular degeneration as mild and multifocal vacuolization of Sertoli cells with desquamated cells containing three or more nuclei (giant cells) inside the seminiferous tubules. We observed a decrease in spermatogonia cells and an increase in spermatocyte cells during the second orchiectomy (late effect), compared to immediate effect (removal of the left testis). This result suggests greater utilization of spermatogonia by the right testis to produce more spermatocytes and reflects a compensatory mechanism aimed at maintaining the sperm production in testicle (Ahn et al., 2021). Hamilton et al. (2018) observed a similar cell abundance within seminiferous tubules after the unilateral orchiectomy agreeing with the data presented in this study, suggesting testicular regeneration.

Unilateral orchiectomy may have caused trauma to the scrotal region, leading to the rupture of the parietal layer of the tunica vaginalis and the tunica albuginea, potentially compromising the testicular parenchyma. This could result in the breakdown of the blood-testis barrier and allows the contents of the seminiferous tubules to enter the bloodstream, which may trigger an acute immune-mediated inflammatory response (Van Camp, 1997; Zhang et al., 1990) and impair sperm production.

Moreover, Santa Inês rams are more resistant to heat stress, as they are adapted to tropical climates. Therefore, an increase of 6^o^C in the testicular temperature achieved in this study was insufficient to promote heat stress in Santa Inês rams, that was described as an adapted breed to high environment temperatures (Correa et al., 2013).

## Conclusion

Testicular insulation in Santa Inês rams, under the experimental conditions of this study, did not cause modifications in the number of seminiferous tubules or the abundance of germ cell within them, therefore the hypothesis was refuted.

The observed decrease in spermatogonia and increase in spermatocytes in the remaining right testis after the first hemi-orchiectomy suggest a physiological compensatory mechanism aimed at preserving sperm production.

## Data Availability

Research data is available in the body of the article.
